# 2,6-Diamino­pyridinium dihydrogen phosphate

**DOI:** 10.1107/S1600536812035489

**Published:** 2012-08-23

**Authors:** Gang Yu

**Affiliations:** a School of Materials Science and Engineering, Shijiazhuang Tiedao University, Shijiazhuang, 050043, People’s Republic of China

## Abstract

In the crystal structure of the title compound, C_5_H_8_N_3_^+^·H_2_PO_4_^−^, N—H⋯O hydrogen bonds, involving the unprotonated amino-group and the NH^+^ group in the pyridinium ring and dihydrogenphosphate O atoms, link the cations and anions. A long chain-like stacking of dihydrogenphosphate anions along the *c-*axis direction is constructed by O—H⋯O hydrogen bonds. Also along the *c-*axis direction, π–π stacking between inversion-related pyridinium rings [centroid–centroid distance = 3.8051 (10) Å] forms columnar stacks of cations.

## Related literature

For functional materials with similar crystal structures and their proton-transfer mechanism, see: Lasave *et al.* (2007 ▶); Morenzoni *et al.* (2007 ▶); Reiter (2002 ▶); Szklarz *et al.* (2011 ▶); Zhang *et al.* (2010 ▶). For the design of similar organic–inorganic functional materials, see: Horiuchi & Tokura (2008 ▶); Zhang & Xiong (2012 ▶).
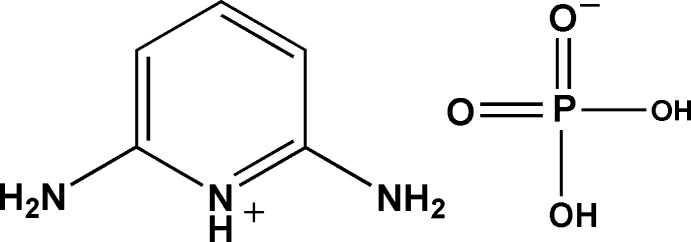


## Experimental

### 

#### Crystal data

C_5_H_8_N_3_^+^·H_2_O_4_P^−^
*M* *_r_* = 207.13
Triclinic, 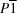

*a* = 7.4821 (4) Å
*b* = 8.1110 (2) Å
*c* = 8.1790 (1) Å
α = 70.811 (10)°
β = 74.980 (14)°
γ = 84.883 (15)°
*V* = 452.77 (3) Å^3^
*Z* = 2
Mo *K*α radiation
μ = 0.29 mm^−1^
*T* = 153 K
0.50 × 0.30 × 0.20 mm

#### Data collection

Rigaku Mercury CCD diffractometer
Absorption correction: multi-scan (*CrystalClear*; Rigaku, 2005 ▶) *T*_min_ = 0.868, *T*_max_ = 0.944
5434 measured reflections
2057 independent reflections
1938 reflections with > σ(*I*)
*R*_int_ = 0.013

#### Refinement

*R*[*F* ^2^ > 2σ(*F* ^2^)] = 0.034
*wR*(*F* ^2^) = 0.090
*S* = 1.17
2057 reflections
133 parameters
4 restraints
H atoms treated by a mixture of independent and constrained refinement
Δρ_max_ = 0.30 e Å^−3^
Δρ_min_ = −0.33 e Å^−3^

### 

Data collection: *CrystalClear* (Rigaku, 2005 ▶); cell refinement: *CrystalClear*; data reduction: *CrystalClear*; program(s) used to solve structure: *SHELXS97* (Sheldrick, 2008 ▶); program(s) used to refine structure: *SHELXL97* (Sheldrick, 2008 ▶); molecular graphics: *SHELXTL* (Sheldrick, 2008 ▶); software used to prepare material for publication: *publCIF* (Westrip, 2010 ▶).

## Supplementary Material

Crystal structure: contains datablock(s) I, global. DOI: 10.1107/S1600536812035489/pk2425sup1.cif

Structure factors: contains datablock(s) I. DOI: 10.1107/S1600536812035489/pk2425Isup2.hkl

Supplementary material file. DOI: 10.1107/S1600536812035489/pk2425Isup3.cml

Additional supplementary materials:  crystallographic information; 3D view; checkCIF report

## Figures and Tables

**Table 1:** Hydrogen-bond geometry (Å, °)

*D*—H⋯*A*	*D*—H	H⋯*A*	*D*⋯*A*	*D*—H⋯*A*
N1—H1⋯O2^i^	0.86	1.90	2.7515 (17)	170
N2—H2*N*2⋯O2^i^	0.85 (2)	2.58 (2)	3.241 (2)	136 (2)
N3—H1*N*3⋯O4^i^	0.87 (2)	2.05 (2)	2.9078 (19)	167 (2)
N2—H1*N*2⋯O1^ii^	0.82 (2)	2.40 (2)	3.0734 (19)	139 (2)
N2—H1*N*2⋯O4^ii^	0.82 (2)	2.56 (2)	3.342 (2)	159 (2)
O1—H1*A*⋯O2^iii^	0.82	1.76	2.5705 (15)	169
O3—H3⋯O4^iv^	0.82	1.73	2.5334 (15)	167
N3—H2*N*3⋯O3	0.85 (2)	2.11 (2)	2.9574 (18)	178 (2)

Symmetry codes: (i) 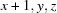; (ii) 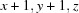; (iii) 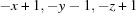; (iv) 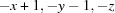.

**Fig. 1.**
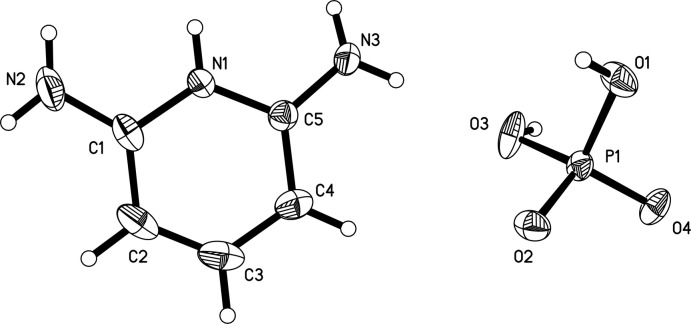
The molecular structure of the title compound drawn with 30% probability displacement ellipsoids for non-H atoms.

## supplementary crystallographic information

### Comment

Recently, explorations for new ferroelectrics of organic-inorganic complexes have attracted extensive attention (Zhang & Xiong, 2012). Among the well known functional materials, KH_2_PO_4_ (KDP) and its analogue crystals have been widely studied from the viewpoint of both their crystal structures and basic physical properties (Lasave *et al.*, 2007; Morenzoni *et al.*, 2007). The O—H···O hydrogen bonds not only support their structural frameworks, but also play critical roles in their properties. The simultaneous displacive deformation of H_2_PO_4_^-^ moieties contributes mainly to spontaneous polarization, together with the protonic order-disorder phenomena. For KDP crystals in an electric field, the collective site-to-site transfer of protons in the O—H···O bonds switches the spontaneous polarization, which is known as the proton-transfer mechanism (Reiter, 2002; Zhang *et al.*, 2010; Horiuchi & Tokura, 2008). As a potential promising strategy, one can imagine that the movements of protons within the hydrogen bonds would be generally advantageous in designing novel ferroelectrics (Horiuchi & Tokura, 2008). Investigation of this type of functional material continues, however, only a few novel crystals have been discovered in recent years. In the present work, a novel complex of the KDP family, 2,6-diaminopyridine phosphate, has been synthesized. Crystal structure analysis (Fig. 1) reveals that O—H···O hydrogen bonds of the H_2_PO_4_^-^ anionic moieties in 2,6-diaminopyridine phosphate assemble into a long chain-like architecture along the *c* axis direction, which is similar to the hydrogen bonds in KDP crystals.

### Experimental

The title complex was synthesized from a mixture of 2,6-pyridinediamine and phosphoric acid with the chemical ratio of 1:1 in aqueous solution. The reaction mixture was stirred for several hours and slowly heated to 45°C yielding a clear solution. After solvent evaporation at controlled temperature for several days, yellow block-shaped crystals were obtained in 96% yield.

### Refinement

H atoms were found in difference Fourier maps. Carbon and oxygen-bound H atoms were subsequently placed in idealized positions with constrained distances of 0.95 Å (C—H) and 0.82 Å (O—H). Nitrogen-bound H atoms were refined subject to distance restraints. U_iso_(H) values were set to either 1.2U_eq_ or 1.5U_eq_ (O—H only) of the attached atom.

### Crystal data

**Table:**

C_5_H_8_N_3_^+^·H_2_O_4_P^−^	*Z* = 2
*M**_r_* = 207.13	*F*(000) = 216
Triclinic, *P*1	*D*_x_ = 1.519 Mg m^−^^3^
Hall symbol: -P 1	Melting point: 396 K
*a* = 7.4821 (4) Å	Mo *K*α radiation, λ = 0.71073 Å
*b* = 8.1110 (2) Å	Cell parameters from 1340 reflections
*c* = 8.1790 (1) Å	θ = 2.7–27.5°
α = 70.811 (10)°	µ = 0.29 mm^−^^1^
β = 74.980 (14)°	*T* = 153 K
γ = 84.883 (15)°	Block, yellow
*V* = 452.77 (3) Å^3^	0.50 × 0.30 × 0.20 mm

### Data collection

**Table:**

Rigaku Mercury CCD diffractometer	2057 independent reflections
Radiation source: fine-focus sealed tube	1938 reflections with > σ(*I*)
Graphite monochromator	*R*_int_ = 0.013
ω and φ scans	θ_max_ = 27.5°, θ_min_ = 2.7°
Absorption correction: multi-scan (*CrystalClear*; Rigaku, 2005)	*h* = −9→8
*T*_min_ = 0.868, *T*_max_ = 0.944	*k* = −10→10
5434 measured reflections	*l* = −10→10

### Refinement

**Table:**

Refinement on *F*^2^	Primary atom site location: structure-invariant direct methods
Least-squares matrix: full	Secondary atom site location: difference Fourier map
*R*[*F*^2^ > 2σ(*F*^2^)] = 0.034	Hydrogen site location: inferred from neighbouring sites
*wR*(*F*^2^) = 0.090	H atoms treated by a mixture of independent and constrained refinement
*S* = 1.17	*w* = 1/[σ^2^(*F*_o_^2^) + (0.0388*P*)^2^ + 0.1286*P*] where *P* = (*F*_o_^2^ + 2*F*_c_^2^)/3
2057 reflections	(Δ/σ)_max_ < 0.001
133 parameters	Δρ_max_ = 0.30 e Å^−^^3^
4 restraints	Δρ_min_ = −0.33 e Å^−^^3^

### Special details

**Table:**

Geometry. All e.s.d.'s (except the e.s.d. in the dihedral angle between two l.s. planes) are estimated using the full covariance matrix. The cell e.s.d.'s are taken into account individually in the estimation of e.s.d.'s in distances, angles and torsion angles; correlations between e.s.d.'s in cell parameters are only used when they are defined by crystal symmetry. An approximate (isotropic) treatment of cell e.s.d.'s is used for estimating e.s.d.'s involving l.s. planes.
Refinement. Refinement of *F*^2^ against ALL reflections. The weighted *R*-factor *wR* and goodness of fit *S* are based on *F*^2^, conventional *R*-factors *R* are based on *F*, with *F* set to zero for negative *F*^2^. The threshold expression of *F*^2^ > σ(*F*^2^) is used only for calculating *R*-factors(gt) *etc*. and is not relevant to the choice of reflections for refinement. *R*-factors based on *F*^2^ are statistically about twice as large as those based on *F*, and *R*- factors based on ALL data will be even larger.

### Fractional atomic coordinates and isotropic or equivalent isotropic displacement parameters (Å^2^)

**Table:**

	*x*	*y*	*z*	*U*_iso_*/*U*_eq_	
N1	1.09610 (18)	−0.12682 (15)	0.27998 (16)	0.0371 (3)	
H1	1.1789	−0.2047	0.3065	0.044*	
N2	1.2828 (3)	0.0630 (2)	0.3140 (3)	0.0656 (5)	
H1N2	1.294 (3)	0.166 (3)	0.304 (3)	0.079*	
H2N2	1.350 (3)	−0.023 (3)	0.354 (3)	0.079*	
N3	0.9398 (2)	−0.34142 (19)	0.2483 (2)	0.0494 (4)	
H1N3	1.044 (3)	−0.400 (3)	0.241 (3)	0.059*	
H2N3	0.852 (3)	−0.368 (3)	0.213 (3)	0.059*	
C1	1.1233 (2)	0.0373 (2)	0.2779 (2)	0.0457 (4)	
C2	0.9892 (3)	0.1617 (2)	0.2384 (3)	0.0615 (5)	
H3C	1.0028	0.2753	0.2361	0.074*	
C3	0.8346 (3)	0.1145 (2)	0.2023 (3)	0.0626 (5)	
H4A	0.7440	0.1984	0.1763	0.075*	
C4	0.8092 (2)	−0.0517 (2)	0.2035 (2)	0.0502 (4)	
H5A	0.7037	−0.0804	0.1788	0.060*	
C5	0.9458 (2)	−0.17538 (19)	0.2426 (2)	0.0376 (3)	
P1	0.44212 (5)	−0.47555 (5)	0.25676 (5)	0.03232 (13)	
O1	0.46973 (19)	−0.65793 (14)	0.39139 (15)	0.0485 (3)	
H1A	0.5193	−0.6456	0.4650	0.073*	
O2	0.39135 (16)	−0.34140 (14)	0.35200 (15)	0.0439 (3)	
O3	0.63300 (16)	−0.4229 (2)	0.12119 (16)	0.0564 (4)	
H3	0.6390	−0.4552	0.0348	0.085*	
O4	0.30132 (14)	−0.50108 (16)	0.16548 (15)	0.0451 (3)	

### Atomic displacement parameters (Å^2^)

**Table:**

	*U*^11^	*U*^22^	*U*^33^	*U*^12^	*U*^13^	*U*^23^
N1	0.0417 (7)	0.0296 (6)	0.0424 (6)	−0.0011 (5)	−0.0130 (5)	−0.0124 (5)
N2	0.0784 (12)	0.0459 (9)	0.0813 (12)	−0.0211 (8)	−0.0222 (10)	−0.0242 (9)
N3	0.0382 (7)	0.0458 (8)	0.0771 (10)	0.0003 (6)	−0.0228 (7)	−0.0301 (7)
C1	0.0575 (10)	0.0331 (7)	0.0434 (8)	−0.0106 (6)	−0.0015 (7)	−0.0140 (6)
C2	0.0734 (13)	0.0313 (8)	0.0683 (12)	0.0015 (8)	0.0017 (10)	−0.0158 (8)
C3	0.0571 (11)	0.0464 (9)	0.0668 (12)	0.0172 (8)	−0.0012 (9)	−0.0102 (8)
C4	0.0384 (8)	0.0548 (10)	0.0524 (9)	0.0092 (7)	−0.0097 (7)	−0.0140 (8)
C5	0.0348 (7)	0.0401 (7)	0.0380 (7)	−0.0006 (6)	−0.0072 (6)	−0.0138 (6)
P1	0.0338 (2)	0.0371 (2)	0.0335 (2)	−0.00135 (14)	−0.01388 (14)	−0.01632 (15)
O1	0.0767 (8)	0.0343 (5)	0.0468 (6)	0.0030 (5)	−0.0286 (6)	−0.0196 (5)
O2	0.0588 (7)	0.0381 (5)	0.0482 (6)	0.0114 (5)	−0.0289 (5)	−0.0223 (5)
O3	0.0419 (6)	0.0955 (10)	0.0429 (6)	−0.0273 (6)	−0.0061 (5)	−0.0329 (7)
O4	0.0328 (5)	0.0688 (7)	0.0477 (6)	−0.0014 (5)	−0.0154 (5)	−0.0322 (6)

### Geometric parameters (Å, º)

**Table:**

N1—C1	1.3586 (18)	C2—H3C	0.9300
N1—C5	1.3601 (19)	C3—C4	1.375 (3)
N1—H1	0.8600	C3—H4A	0.9300
N2—C1	1.350 (3)	C4—C5	1.389 (2)
N2—H1N2	0.822 (18)	C4—H5A	0.9300
N2—H2N2	0.848 (19)	P1—O4	1.5028 (10)
N3—C5	1.336 (2)	P1—O2	1.5050 (10)
N3—H1N3	0.874 (17)	P1—O3	1.5584 (12)
N3—H2N3	0.852 (17)	P1—O1	1.5608 (12)
C1—C2	1.379 (3)	O1—H1A	0.8200
C2—C3	1.378 (3)	O3—H3	0.8200
			
C1—N1—C5	123.93 (14)	C4—C3—H4A	118.7
C1—N1—H1	118.0	C2—C3—H4A	118.7
C5—N1—H1	118.0	C3—C4—C5	118.03 (17)
C1—N2—H1N2	110.8 (18)	C3—C4—H5A	121.0
C1—N2—H2N2	120.4 (18)	C5—C4—H5A	121.0
H1N2—N2—H2N2	128 (3)	N3—C5—N1	116.91 (13)
C5—N3—H1N3	117.6 (14)	N3—C5—C4	124.57 (15)
C5—N3—H2N3	116.4 (15)	N1—C5—C4	118.52 (14)
H1N3—N3—H2N3	120 (2)	O4—P1—O2	115.15 (6)
N2—C1—N1	115.83 (16)	O4—P1—O3	110.88 (6)
N2—C1—C2	126.02 (16)	O2—P1—O3	107.55 (7)
N1—C1—C2	118.14 (17)	O4—P1—O1	106.11 (7)
C3—C2—C1	118.83 (16)	O2—P1—O1	110.22 (6)
C3—C2—H3C	120.6	O3—P1—O1	106.64 (8)
C1—C2—H3C	120.6	P1—O1—H1A	109.5
C4—C3—C2	122.54 (17)	P1—O3—H3	109.5
			
C5—N1—C1—N2	−178.37 (15)	C2—C3—C4—C5	0.0 (3)
C5—N1—C1—C2	1.1 (2)	C1—N1—C5—N3	179.29 (15)
N2—C1—C2—C3	179.19 (19)	C1—N1—C5—C4	−1.4 (2)
N1—C1—C2—C3	−0.2 (3)	C3—C4—C5—N3	−179.93 (17)
C1—C2—C3—C4	−0.3 (3)	C3—C4—C5—N1	0.8 (2)

### Hydrogen-bond geometry (Å, º)

**Table:**

*D*—H···*A*	*D*—H	H···*A*	*D*···*A*	*D*—H···*A*
N1—H1···O2^i^	0.86	1.90	2.7515 (17)	170
N2—H2*N*2···O2^i^	0.85 (2)	2.58 (2)	3.241 (2)	136 (2)
N3—H1*N*3···O4^i^	0.87 (2)	2.05 (2)	2.9078 (19)	167 (2)
N2—H1*N*2···O1^ii^	0.82 (2)	2.40 (2)	3.0734 (19)	139 (2)
N2—H1*N*2···O4^ii^	0.82 (2)	2.56 (2)	3.342 (2)	159 (2)
O1—H1*A*···O2^iii^	0.82	1.76	2.5705 (15)	169
O3—H3···O4^iv^	0.82	1.73	2.5334 (15)	167
N3—H2*N*3···O3	0.85 (2)	2.11 (2)	2.9574 (18)	178 (2)

Symmetry codes: (i) *x*+1, *y*, *z*; (ii) *x*+1, *y*+1, *z*; (iii) −*x*+1, −*y*−1, −*z*+1; (iv) −*x*+1, −*y*−1, −*z*.
